# Methodological aspects of testing vestibular evoked myogenic potentials in infants at universal hearing screening program

**DOI:** 10.1038/s41598-019-53143-z

**Published:** 2019-11-21

**Authors:** Luca Verrecchia, Niki Karpeta, Magnus Westin, Ann Johansson, Sonny Aldenklint, Krister Brantberg, Maoli Duan

**Affiliations:** 1 Audiology and Neurotology Unit, Ear Nose and Throat Patient Area, Trauma and Reparative Medicine Theme, Karolinska University Hospital, Stockholm, Sweden; 2 Division of Ear, Nose and Throat Diseases, Dept of Clinical Science, Intervention and Technology, Karolinska Institutet, Stockholm, Sweden

**Keywords:** Neurology, Medical research

## Abstract

Motor development in infants is dependent upon the function of the inner ear balance organ (vestibular organ). Vestibular failure causes motor delays in early infancy and suboptimal motor skills later on. A vestibular test for newborns and infants that is applicable on a large scale, safe and cost effective is in demand in various contexts: in the differential diagnosis of early onset hearing loss to determine forms associated with vestibular failure; in early hearing habilitation with cochlear implant, indicating the vestibular predominant side; and in the habilitation of children affected by motor skill disorders, revealing the contribution of a vestibular failure. This work explored the feasibility of cervical vestibular evoked myogenic potentials (VEMP) in conjunction with newborn universal hearing screening program. VEMP was measured after the hearing tests and was evoked by bone-conducted stimuli. Moreover, stimulus delivery was regulated by neck muscle activity, with infants rested unconstrained in their parents´ arms and with the head supported by the operator´s hand. This VEMP protocol showed a high level of feasibility in terms of test viability and result reproducibility. VEMP integrated into the newborn hearing screening program may represent a practical method for large-scale assessment of balance function in infants.

## Introduction

The inner ear balance organ (i.e., the vestibular organ) plays a fundamental role in motor development in newborns and infants. An early vestibular assessment is currently in demand in various clinical contexts and could be made possible by advancements in the clinical testing of vestibular function in children. Children with sensorineural hearing loss (SNHL) are more often affected by vestibular dysfunction (VD)^1^. VD in newborns and infants, particularly in the form of bilateral vestibular failure, is an independent factor for delayed and suboptimal motor proficiency^2,3^. Moreover, VD affects reading acuity in school age children^4^ and it is associated with unprotected falls with head trauma^5^.

An early vestibular assessment may contribute to the prognostic stratification of motor disabilities in children, specifically in those affected by syndromic SNHL or those affected by SNHL due to congenital viroses (citomegalovirus, rubella, etc.). These children may show early onset SNHL, vestibular impairment and neurological disorders. Vestibular assessment may reveal motor disorders related to vestibular failure and, in this way, could have positive prognostic relevance: vestibular-related motor delays are expected to reach acceptable functional levels by school age^6^ and they may be enhanced by specific vestibular habilitation programmes^7^.

Testing vestibular function in infants may contribute to the early diagnosis of congenital or early onset SNHL. In a clinical context in which up to 35% of congenital SNHL patients receive no specific diagnosis^8^, a documented vestibular failure may drive the diagnosis towards SNHL associated with vestibular failure especially prevalent in inner ear malformations^9^, Usher´s syndrome^10^, congenital citomegalovirus infection (cCMV)^11,12^ and neuropathies^13^.

An early vestibular assessment has also become mandatory in terms of cochlear implantation (CI). CI is the best solution for hearing and speech habilitation in severe childhood SNHL. However, this procedure is thought to alter the vestibular function^14–18^. In a target population, SNHL infants, in which pre-existing vestibular dysfunction is probable, the CI would represent an added risk of developing vestibular failure^19^.

Given these premises, a vestibular test for newborns and infants that is applicable on a large scale, safe and cost effective is worth consideration.

We studied the feasibility of a vestibular test, vestibular evoked myogenic potentials (VEMP) integrated into the universal newborn hearing screening program. VEMP has already been applied in newborns and infants, but in limited cohorts^20,21^. This study focused on the methodological aspects of large-scale VEMP testing with a specific protocol based on bone-conducted stimulation and on recordings under controlled neck muscle activity levels.

## Material

The target population was composed of children included in the regional newborn hearing screening program^22^. Hearing screening was conducted at 8 different birth centres, covering 98,7% of newborns (data from 2016, 30036 newborns). According to the programme, newborns failing two consecutive transient-evoked otoacoustic emission tests (TE-OAE) are referred at four weeks of life for a second step that consists of a third TE-OAE, and if there is no response, automatic auditory brainstem responses (aABR) are measured. In 2016, 3,2% of the total population of newborns were referred for second step hearing screening at our tertiary university hospital audiological service. Failed responses in both procedures indicated hearing loss worse than 30 dB HL. Infants failing the second step were further referred for a diagnostic evaluation, consisting of a clinical ABR and medical examination. Newborns affected by conditions at high risk of developing congenital or early onset SNHL (severe prematurity, bacterial/viral meningitis, severe newborn jaundice, severe hypoxic ischaemic encephalopathy, congenital virus infections, family history of SNHL, foetal alcoholic syndrome, intraventricular haemorrhages, periventricular leukomalacia, genetic syndromes associated with SNHL) were included in the universal hearing screening program as at-risk group and were referred directly to the second step.

VEMP testing was planned in children who were referred to the second step or clinical evaluation, both for logistical reasons (it was hard to introduce an experimental method on a large scale at birth centres) and also to address VEMP in a smaller cohort of children with a higher SNHL prevalence and, consequently, a higher incidence of VD.

A retrospective analysis of the hearing screening program in the Stockholm region resulted in an SNHL prevalence of 0.3% of newborns. Assuming that half of these newborns had vestibular loss^1^, a representative sample size would be no fewer than 45 children (power analysis based on alpha of 0.05 and a beta of 0,80).

We enrolled 50 infants in a period of 14 months. The study was performed in accordance with the Declaration of Helsinki guidelines and was approved by the Stockholm regional ethics committee (protocol number 2015/1296-31/2). Recruitment was conducted after obtaining informed written consent from the parents. We excluded critically ill infants. The collected sample represented 60% of the eligible candidates for VEMP testing, as 40% of the parents declined to participate in the study. VEMP was recorded on both sides, except in one case. A total of 99 ears were tested in 50 children.

## Method

### Test specifications

Recording VEMP at the second step hearing screening or clinical evaluation permitted us to measure VEMP in continuity with hearing testing. In fact, it was relatively easy to convert a commercially available ABR device for VEMP testing. We used the signal averager Eclipse EP 25 with the VEMP module (Interacoustic A/S, Middelfart, Denmark). This device has software modules dedicated to ABR and a panel of VEMP test protocols.

The VEMP measured in this study was cervical VEMP (cVEMP), and the method to measure this VEMP is currently regulated in adults by expert guidelines^23^. cVEMP is a myogenic potential induced by vestibular stimulation that is easily evoked by loud sound impulses to the ear. These potentials are recorded at the ipsilateral sternocleidomastoideus muscle (SMC) with surface electromyography (EMG), and they appear as short-latency positive-negative deflections of the basal EMG recording. Collecting and averaging 100-250 sweeps is enough to obtain stable responses. cVEMP is generally studied in terms of amplitude analysis, considering that most peripheral vestibular disorders do not affect the response latency. VEMP amplitude is dependent upon four parameters: the vestibular function, the muscle activity, the stimulus intensity and the stimulus configuration. Given the inhibitory nature of the sacculo-collic myogenic reflex at the basis of the cVEMP, the test requires sustained SMC muscle tone, obtained in adults by voluntary head elevation in supine position or by protracted head turns in sitting position^24^. Variability in muscle activation levels is common and can affect cVEMP reproducibility. In fact, a threshold effect is observed at EMG levels under 50 µVolts, and a linear amplitude increase up to 400 µVolts. Upon these EMG values it is expected an amplitude saturation effect. Hence, it is crucial to maintain muscular activity within the range of the linear amplitude/EMG to obtain reproducible results. By scaling the response amplitude over the prestimulus EMG level and by using standardized stimulus configuration and intensity, the cVEMP amplitude can be used for the study of the peripheral vestibular function. In children, the execution of the test is complicated by poor compliance. Sustained neck muscle activation cannot be demanded of infants, and the frequent presence of vernix in the external canal or effusion in the middle ear may affect the acoustic admittance to air-conducted stimuli. To compensate for these drawbacks, we applied a stimulation protocol based on:

- EMG-driven stimulus delivery algorithm: the device permitted the monitoring of the EMG at the recording site and permitted also to control the stimulus delivery by this EMG level. According to the factory specifications, the EMG level at recording point was sampled repetitively over intervals of 100 ms and averaged as the root mean square (RMS) of rectified EMG. For this study, we maintained the factory settings of stimulus delivery within an EMG reference level of 50–150 µVolts. Once EMG resulted within this reference interval, the device operated a new RMS averaging of rectified EMG over an interval of 100 ms (prestimulus EMG sample) followed by stimulus delivery. The EMG recording was continued up to 80 ms after stimulus and the EMG values within the interval −20 ms, +80 ms were processed as VEMP recording window. This operational algorithm was repeated for each sweep collected in the recording session (trial).
- Bone-conducted stimuli instead of sound stimuli: the stimuli were applied on the mastoid bone and consisted of a tone burst vibration delivered by a Radioear B71 bone vibrator device (Radioear Corp, New Eagle, PA, USA) at 500 Hz and 50 dB nHL (119 dB FL) with a 2 ms rise-plateau-fall configuration and a stimulation rate of 5.1/sec.

Recordings were performed unilaterally. Two inverting electrodes were added to the belly of the two SCMs: the non-inverting electrode was on the manubrium sterni, and the ground electrode was on the forehead. The skin was prepared with gentle abrasion to maintain an electrode impedance under 10 kΩ. The signal was processed by amplification (gain: 2000) and was bandpass filtered (10–750 Hz) within the recording window from −20 to 80 ms. At least 120 sweeps were collected, with a maximum of 200, for each trial.

### Conduction of the test

VEMP was recorded by four audiologists trained in paediatric audiological tests after the scheduled aABR at the second step or diagnostic ABR. During VEMP testing, the child was supine in the parent’s arms with the head supported on the examiner’s hand and was awake enough to generate neck muscle activity. The bone transducer was maintained in contact with the skin of the mastoid region by the operator with lateromedial digital pressure and was placed above an imaginary antero-posterior line crossing the ear canal. SMC activity could be monitored on a screen as EMG levels at the recording site. The examiner, modulating the child´s head support, could change the SCM activity and the corresponding EMG level on screen, probably by acting on the neck muscles myotatic reflex. In older infants, muscle tonus could have been influenced by voluntary activity, especially in cases of agitation. The examiner was instructed to maintain the SMC activity level as stable as possible within an EMG window of 50 to 150 μVolts, adjusting the head support in response to the EMG values on screen (Fig. 1 in Supplementary Information shows a schematic representation of the VEMP recording settings). The duration of the trials was as long as the child could allow, with a primary goal of 120 collected responses and a secondary goal of 200 collected responses. In cases of insufficient sweep collection or scarce wave reproducibility, VEMP recording was repeated in further trials, as long as the child permitted test prolongation. Testing was generally completed within 90–120 seconds per side. Protracted crying, scarce wakefulness or agitation were common reasons for the interruption of the test before the collection of 120 responses.

### Data collection and statistical analysis

For each subject, we collected demographic data (age in months and gender), data on diagnosis and comorbidity and data regarding whether the subject was referred to the second step because he or she failed the newborn hearing screening or because he or she belonged to the at-risk group.

Moreover, for each tested ear, the following data were collected:

1. Hearing loss (HL): the presence/absence of HL was defined by a pathological response at AABR or ABR, corresponding to an HL of ≥35 dB HL for air-conducted stimuli.
2. VEMP response: VEMP was defined as positive-negative EMG deflection with a latency of 12–17 ms for the first peak (p1) and 20–25 ms for the second peak (n1) after stimulus. The response had to resemble the typical response obtained in adults by AC stimulation, which is highly indicative of ipsilateral vestibular function^25^. VEMP identification was based on morphological analysis, as commonly conducted in adults. The analysis was accomplished by the senior author (KB), who was blinded to the test parameters and clinical conditions.
3. Muscle activity: the device could calculate the mean (SD) in µVolts of the prestimulus EMG samples collected in the trial. This mean value was named prestimulus EMG and used as a marker of muscle activation during testing.
4. VEMP amplitude: measured as the N1-P1 interpeak EMG difference in µVolts. A scaled amplitude over the prestimulus EMG was also given. The amplitude was furthermore expressed in terms of the asymmetry ratio (AR), and the equivalent was calculated also for the corrected amplitude (corrAR). AR was defined as the absolute value of the ratio of the amplitude difference between the sides over the sum. For recordings with no apparent response, an n1-p1 interval was calculated arbitrarily with EMG values measured at 13 ms and 23 ms after stimulus.
5. VEMP latency: the latency of the first peak, n1, and the second peak, p1, were given in milliseconds (ms).
6. Completion grade: measured as the number of collected responses (sweeps) for each trial. A cut-off of 120 sweeps defined test completion.

When there were two or more recording trials, the trial with higher completion grade and the best VEMP waveforms was considered.

Summary statistics were provided for both demographic and test variables. These parameters were used as control variables for the analysis of two feasibility indicators, the completion rate (CR) and the response rate (RR).

CR was the proportion of tested ears with at least 120 collected sweeps; it was a marker of test completion in the sample and, consequently, an index of test viability on a large scale in infants. CR was analysed with respect to diagnosis, hearing loss and the prestimulus EMG cut-offs. Non-parametric tests (χ^2^ test) were applied to study the association between CR and those determinants, whereas parametric tests (Mann-Whitney U-test) were applied to study the relation between CR and the number of collected sweeps, prestimulus EMG, and response amplitude.

The second outcome, RR, was computed as the proportion of the tests in which it was possible to identify a characteristic VEMP response. RR represented an index of test reproducibility. RR was studied with respect to diagnosis, HL, CR, prestimulus EMG cut-offs with non-parametric analysis. Receiver operating curve (ROC) analysis was performed to define the amplitude cut-off for the response identification.

Statistical significance was stated at p < 0.05.

## Results

### Descriptive statistics

The children showed an equal distribution across genders (M: 52%; F: 48%) and were tested at a mean age of 2.3 ± 1.9 months (median: 2; range: 1–6; mode: 1).

### Hearing loss and diagnosis

HL was diagnosed in 18 (36%) subjects. HL was diagnosed in 10 of 26 infants (38.4%) who failed the newborn hearing screening and in 8 of 24 infants (33%) included in the at-risk group. It was possible to establish the aetiology of hearing loss in four infants in the first group (Table 1). HL in the at-risk group was directly related to the background diagnosis or to an associated middle ear effusion, for example, in the case of Down syndrome or cleft palate.

**Table 1:** Case distribution in diagnostic groups and type of hearing loss.

Group	Diagnosis	Unil HL	Bil HL	No HL
Refer	cCMV		1	
	Connexin 26 mutation		1	
	SOM	1		4
	VIII Nerve hypoplasia		1	
	Not defined	5	1	12
Total (%)	26 (52)	6 (23)	4 (15,4)	16 (61,6)
Risk group	Down Syndrome	1	1	2
	Leber´s Amaurosis			1
	Syndromic n.s.	1		2
	Preterm	1		3
	HIE		1	6
	Sepsis/meningitis			2
	Cleft palat	2	1	
Total (%)	24 (48)	5 (20,8)	3 (12,5)	16 (66,7)

Uni/BIl/No HL: unilateral/bilateral/absent hearing loss. Acronyms: cCMV: congenital cytomegalovirus infection; SOM: serous otitis media; syndromic n.s.: syndromic phenotype, not specified; HIE: hypoxic ischemic encephalopathy. Within brackets the proportion in percentage referred to the whole group (column diagnosis) or diagnostic subgroup (columns HL).

### Amplitude and latency

**Figure 1:**
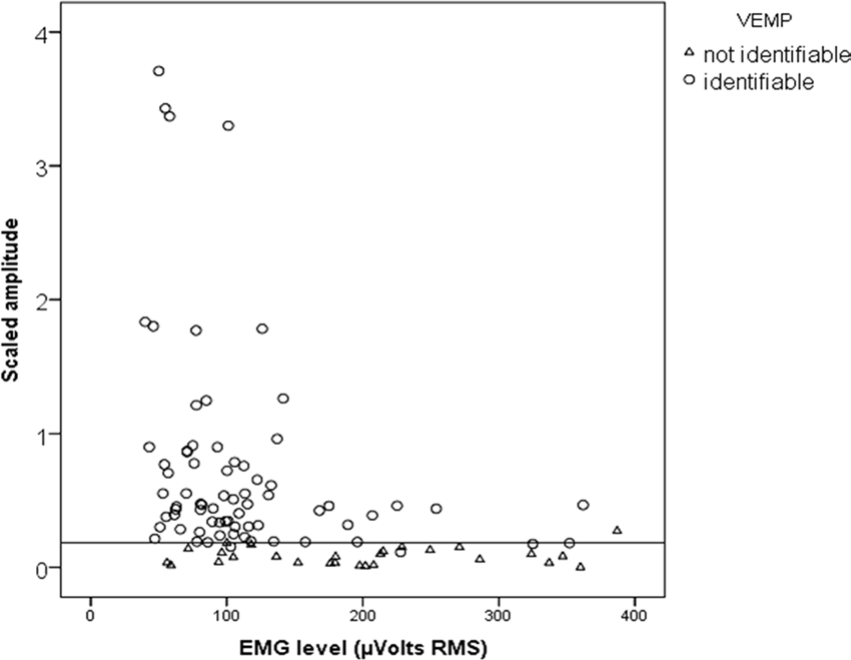
Effect of the prestimulus EMG on the VEMP scaled amplitude: at EMG over 150 µVolts the amplitude is generally depressed and closer to the detection threshold (horizontal line: 0,18 value scaled amplitude).

Amplitudes and latencies were analysed in the identifiable responses. The absolute amplitude was characterized by a skewed distribution with a median value of 44,1 µVolts (IQR: 30,0–77,2; min: 11,3; max: 285,1). The corrected amplitude distribution was also skewed, with a median value of 0,46 (IQR: 0,30–0,79; min: 0,11; max: 3,7). Amplitude and corrected amplitude did not correlate with age, gender, latency, prestimulus EMG, or grade of completion and did not differ significantly based on diagnosis or HL. The two amplitude parameters correlated strongly with themselves (Pearson’s r = 0,90, p < 0,001). Moreover, by stratifying the scaled amplitudes over the prestimulus EMG, it was possible to identify an EMG cut-off level of approximately 150 µVolts upon which the VEMP response systematically subsided (Fig. 1). The AR had a median value of 0,25 (IQR = 0,13–0,46; min = 0; max = 1); additionally, the corrAR had a mean = 0,32 (±0,24; min = 0; max = 1). Restricting the AR analysis to the 34 subjects with clearly identifiable VEMP responses on both sides, the AR was 0,25 (±0,16; min: 0,1; max: 0,58), and the corrAR was 0,26 (±0,19; min: 0; max: 0,68).

The peak latencies had a mean P1 value = 14,1 ms (±1,3; min: 11,3, max: 18,3) and a mean N1 value = 22,3 ms (±2,1; min: 18,3, max: 26,7). Latencies did not correlate with any of the other clinical parameters, except the P1 latency, which correlated weakly with age (Spearman’s ρ = −0,349, p = 0,008). We also modelled this relation as a linear regression, P1(ms) = −0,296 * (months) +14,8 (R^2^ = 0,31, p = 0,021).

### VEMP Completion rate (CR)

**Figure 2:**
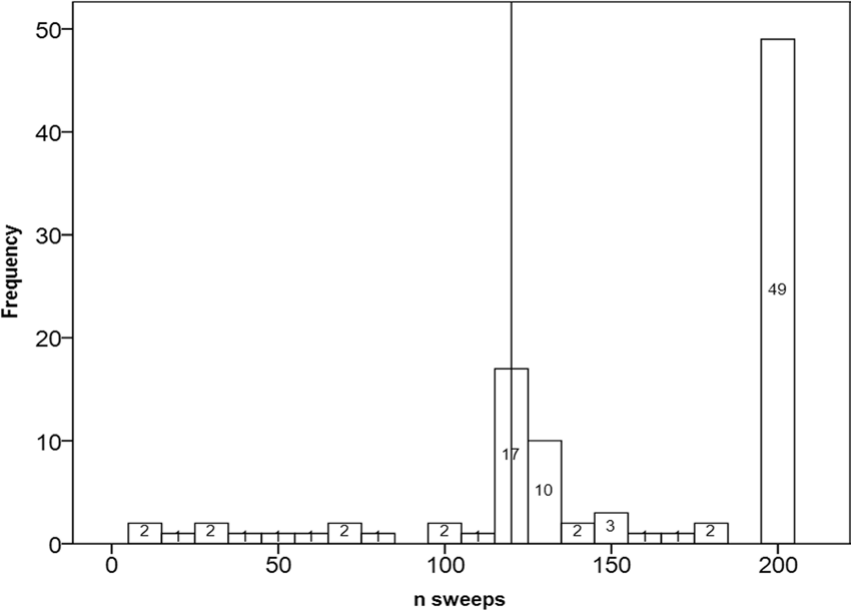
Histogram showing the sample distribution (ears) related to the number of completed recordings. Vertical line points out the target level for test completion (120). Nearly half of the subjects reached the maximum default of 200 collected sweeps.

In 86 of the 99 tested ears (86,8%), it was possible to complete VEMP testing by collecting at least 120 sweeps. In nearly half of the tested ears (49/99), sampling reached the default maximum of 200 sweeps. In seven subjects, we could only complete the test on one side. In four subjects, VEMP testing was not completed on either side. Figure 2 shows the case distribution for CR classes.

**Figure 3:**
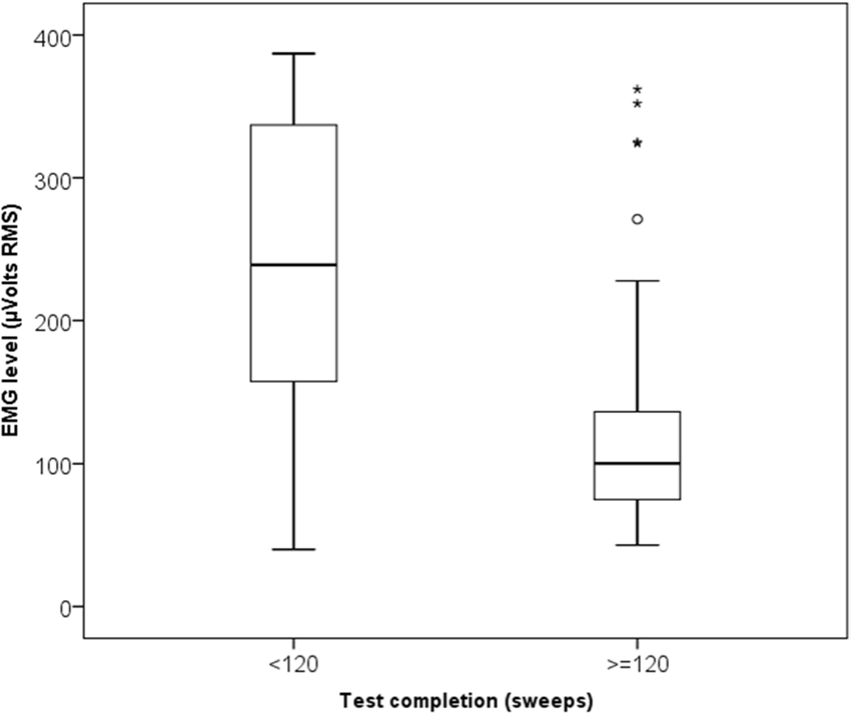
Box plot indicating the difference in prestimulus EMG between the completed and not completed tests. o: outlier; *extremes. Difference statistically significant at p < 0,01.

Looking at the factors affecting the CR, the prestimulus EMG was the only statistically significant factor. As shown in Fig. 3, incomplete VEMP recordings were associated with significantly higher prestimulus EMG. Despite the application of a recording protocol limited by the detection of EMG at recording point within the reference interval of 50–150 µVolts, several VEMP responses were recorded at prestimulus EMG outside that interval. Limiting the analysis to the recordings with the prestimulus EMG within the detection interval, 2/65 incomplete tests resulted (3%, CR = 97%); the proportion of incomplete tests was significantly higher outside the interval: 25% were under the cut-off of 50 µVolts (1/4 tests) and 37% were over 150 µVolts (11/30).

### VEMP response rate

**Figure 4:**
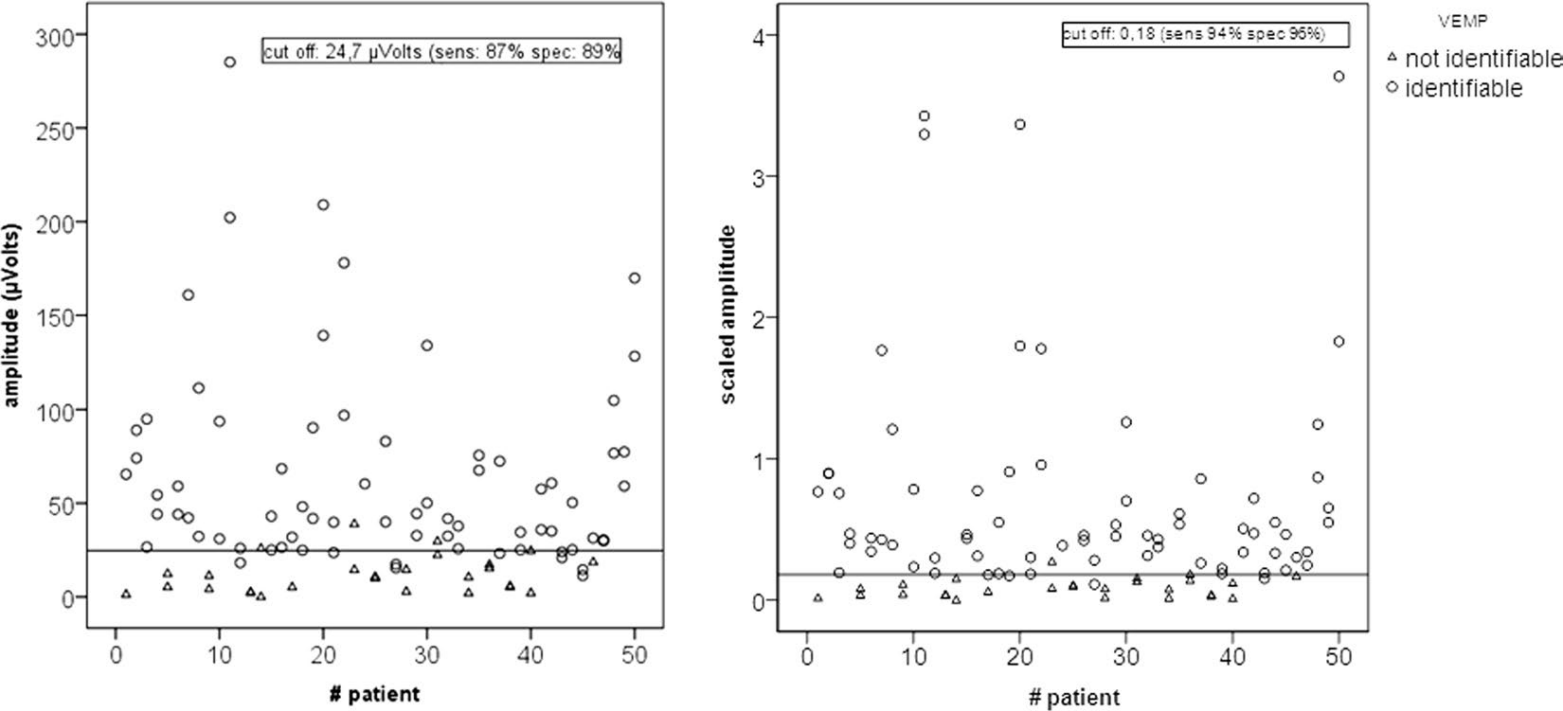
Distribution of identifiable vs not identifiable VEMP for subject (x axis) and amplitude (y-axis left) or scaled amplitude (y-axis right). The majority of the non-identifiable VEMP had an amplitude less than 24,7 µVolts or scaled amplitudes less than 0,18. The diagnostic precision of these two cut offs is provided.

A VEMP response was identified in 72 (73%) of the tested ears. The best amplitude cut-off for VEMP identification was 24.7 μVolts (sensitivity of 87% and specificity of 89%). The corresponding cut-off value of 0.18 obtained on the scaled amplitudes was able to separate the identifiable responses from non-identifiable responses with a sensitivity of 94% and a specificity of 96% (Fig. 4).

Three parameters could significantly affect the RR: CR, HL and the prestimulus EMG.

A higher RR was present in completed tests than uncompleted tests (78,8% vs 35,7%, χ^2^ test: 11,2. p = 0,02), in ears with normal hearing than in those showing HL (78,4% vs 56%, χ^2^ test: 4,72. p = 0,03) and in tests with prestimulus EMG within the reference interval than in those outside this range (86,1% vs 47%, χ^2^ test: 17,1. p < 0,001). Finally, correcting for all three determinants (no HL, CR ≥ 120 and prestimulus EMG within 50–150 µVolts; ideal conditions were fulfilled in 48 ears), the response rate increased from 73% to 91.5%.

#### RR, diagnosis and HL

The relationship between HL, HL diagnosis and RR is summarized in Table 2. To reduce the effects of confounding factors, this analysis was conducted including only the cases with ideal testing conditions (prestimulus EMG within 50–150 µVolts and CR ≥ 120; 63 ears). VEMP responses were absent in cCMV^11^ and nerve hypoplasia cases^13,26^, whereas they were present in cases of connexin 26 gene mutation^27^. Interestingly, in the at-risk group, VEMP was present in all cases of hypoxic encephalopathy and in severe prematurity, in which the hearing was also unaffected. VEMP was also found, with hearing, in cases of meningitis/sepsis, indicating that these illnesses had spared the inner ear structures^9^. A few examples of VEMP recordings are given in the Supplementary Information. VEMP tended to have a concordant pattern with hearing result at screening: 43/54 identifiable VEMPs were found in ears with normal hearing, and no identifiable VEMPs were found in six of the nine ears with HL. The odds ratio of a VEMP/hearing concordance was 2,56: it was two and half-fold more probable to find concordant VEMP and hearing response than the opposite.

**Table 2:** Sample distribution (ears) according to VEMP, hearing loss and test agreement between VEMP and hearing screening.

Group	Diagnosis	VEMP+	HL	VEMP−	HL	Test agreement
Refer	CMV	0	0	2	2	100%
	Connexin 26 mutation	2	2	0	0	0%
	SOM	5	1	0	0	80%
	Nerve Hypoplasia + IP	0	0	2	2	100%
	Not defined	15	4	2	1	70,5%
Total (%)	28 (44%)	22	5	6	5	
Risk group	Down Syndrome	4	0	1	0	80%
	Leber´s Amaurosis	1	0	0	0	100%
	Syndromic n.s.	5	1	0	0	80%
	Preterm birth	5	1	0	0	80%
	Hypoxic Encephalopaty	11	2	0	0	82%
	Sepsis/meningitis	3	0	0	0	100%
	Palatoschisis	3	2	2	1	40%
Total (%)	35 (56%)	32	6	3	1	

VEMP+: ears with identifiable VEMP; VEMP−: ears with no identifiable VEMP; HL: ears with hearing loss; test agreement: proportion of ears with concordant VEMP/hearing level, intended as ears showing identifiable VEMP and normal hearing or absent VEMP and HL. Refer to Table 1 for other abbreviations.

## Discussion

This study confirmed the high feasibility of VEMP integrated in the newborn hearing screening program. VEMP measurement, introduced in the second step of hearing screening or after the clinical ABR, could be completed in most of the tested ears (86%) and in up to 97% of recordings with prestimulus EMG within reference. Further, more than three quarters of the tests resulted in an easily identifiable VEMP response, up to 91.5% when the test was conducted under ideal clinical conditions. However, the study showed also that VEMP reproducibility was prone to procedural bias, mainly the regulation of EMG levels during recording. Other tests have been proposed for the screening of vestibular function in children. A valid alternative to VEMP is the video head impulse test (vHIT), which is based on computerized eye tracking systems. It studies the efficacy of the vestibular ocular reflex in gaze holding during passive head turns^28^. In our experience, the vHIT may be difficult to conduct in infants younger than 4–5 months old, given the difficulty they have in sustaining visual fixation on a target, especially during head movements. Moreover, the VOR gain in the first months of life is naturally depressed. These two factors can reduce the value of the vHIT for vestibular testing in infants. Conversely, VEMP recording can be easily integrated with hearing screening programmes, allowing VEMP measurement to be performed in continuity with the ABR testing.

In healthy adults, the corrected amplitude of cVEMP evoked by submaximal AC stimuli (133 dB SPL) has shown a wide variability (mean: 1,2; range: 0–4,5)^29^; in this cohort, the median corrected amplitude was lower at 0,46 (min: 0,1-max: 3,7), anyway obtained with less intensive BC stimuli (119 dB FL). Looking at the distribution of the parameter corrAR, a value over 0,64 (corresponding to a corrected amplitude 4,6-fold larger on one side than the other) could be considered outside a normal variability range. VEMP latencies were like those expected in adults, with a possible trend of P1 shortening with age at a rate of −0,35 ms/month.

A screening test, in addition to its diagnostic properties, has to fulfil specific methodological requirements: it should be inexpensive, easy to administer, not harmful, and cover a population in which the target disease occurs with high incidence^30^. VEMP conducted at newborn hearing screening accomplished these criteria: converting a commercially available ABR device used for hearing screening to measure VEMP maximized the cost containment and viability of a large-scale vestibular test in infants. Moreover, VEMP could be measured in the most tested ears with a CR of up to 97% under optimized conditions. Finally, VEMP was added at the second step hearing screening or at the clinical ABR, in which a high prevalence of the target condition, VD, is expected (estimated to occur in 1/20 children).

VEMP has been recently introduced in a large multicentre national study as a second-level vestibular assessment for all SNHL newborns detected during the hearing screening program^31^. Dealing with the same idea, the present study aimed, instead, to integrate VEMP into the hearing screening program for all children tested after the first step of hearing testing. In this way, it was possible to enlarge the study to normal hearing infants (the refer ones at the first step and dismissed during the following steps). This approach permitted to show the negative effect of HL on the RR, most likely due to a higher prevalence of vestibular failure in the HL group. Moreover, integrating VEMP in hearing screening permitted the study of vestibular function in specific cohorts of infants regardless of hearing loss. For example, it was possible to demonstrate that conditions such as preterm birth and hypoxic encephalopathy, as well as syndromes such as Down syndrome, had a limited effect on vestibular function (Table 2).

### VEMP response and its determinants

This study confirmed, in part, the literature trends regarding the aetiology of vestibular failure in children: we obtained, for example, poor VEMP responses in individuals with cCMV^11^ and nerve hypoplasia^13,26^. However, we have also obtained unexpected results, such as VEMP and hearing maintenance in meningitis/sepsis^9^.

VEMP was not retrieved in 9 of the 63 recordings conducted under ideal test conditions (CR ≥120 and prestimulus EMG within 50–150 µVolts). Whereas 4 of these cases were associated with diagnoses in which it is highly probable to find vestibular failure (CMV and nerve hypoplasia), 5 could not be explained by the clinical background, and 3 were in ears with normal hearing levels. To our knowledge, the presence of isolated vestibular loss in the presence of normal hearing has never been reported in infants. Further investigation is needed to confirm the presence of isolated vestibular loss in children.

VEMP was not altered by middle ear disorders. In fact, VEMP responses could be retrieved in all cases diagnosed with SOM. Moreover, there was no significant difference in the RR between conditions at high risk of high acoustic impedance (Down syndrome and cleft palate; 21 ears) and the remaining cases (61,9% for the formers and 75,69% for the latter, Fisher’s exact test, p = 0,16). VEMP by BC, in other words, bypassed the elevated acoustic impedance often found in newborns´ ears.

According to a recent systematic review^32^, VEMP failure—at least on one side—has been demonstrated in 13% to 84% (weighted prevalence: 32%) of paediatric SNHL cases. Comparing these data to those of our cohort (restricted to the 63 recordings under ideal conditions and excluding 4 cases at high risk of elevated acoustic impedance), a subgroup of 11 ears with SNHL was found, four of which were unable to reproduce VEMP. This result corresponded to VEMP failure coupled to SNHL at a rate of 36%, in line with the literature trends.

### Preliminary considerations of VEMP validity as a vestibular screening test

Determining the validity of VEMP, in terms diagnostic accuracy for vestibular impairment in infants, was beyond the scope of this study, which had the aim to explore the test feasibility. However, some preliminary considerations can be drawn. VEMP, as a screening procedure, must generate minimal false negative results and should be integrated into a multi-step diagnostic protocol for the control of the false positive rate.

Regarding false positive responses (unidentifiable VEMP responses in cases of normal vestibular function), some concerns were raised in this pilot study. The test results, both in terms of CR and RR, were significantly affected by the prestimulus EMG. The reproducibility of cervical VEMP is optimal in a specific EMG range. Outside of this range, the amplitude may become markedly depressed^24^. This finding has been demonstrated in adults under voluntary neck muscle contraction. The same pattern was demonstrated in this cohort, with a clear amplitude depression occurring with prestimulus EMG outside the reference interval (Fig. 1). In addition, the recording protocol, based on EMG controlled stimulus delivery, appeared effective in filtering the recordings at insufficient prestimulus EMG, but the same cannot be said for those recorded at excessive prestimulus EMG (over 150 µVolts and, in some cases, up to 350 µVolts). Further technical development in the EMG stimulus delivery protocol in infants is required to abate the false positive results due to excessive muscle activity during recording. Furthermore, in these children, we obtained sustained muscle tonus by modulating head support, most likely acting on the stretch reflex of the neck muscles. It is unclear whether the way a muscle contraction is obtained, i.e., voluntarily in adults or by reflex in infants, could differently influence the VEMP response pattern. Reflexive SMC activation has been used to measure VEMP in newborns, for example, stimulating the rooting reflex in head turned newborns^21^. This study has demonstrated that VEMP obtained with reflexive SMC activation could be achieved in a large sample of infants with high reproducibility.

Regarding the false negative responses, they are identifiable VEMP responses in the presence of a real vestibular deficit. A weak to moderate correlation between different vestibular testing methods has been observed in children^1^. In adults, the combination of normal cVEMP and a pathologic caloric response is common in superior divisional vestibular paresis, usually documented in vestibular neuronitis^33^. VEMP is also spared in the majority of acquired progressive bilateral vestibulopathies^34^, in which other vestibular test results are commonly depressed. Thus, a discrepancy between VEMP and other vestibular tests is possible and evident in cases of partial or progressive vestibular loss; however, it seems less probable to elicit a VEMP response in cases of extensive vestibular damage/vestibular areflexia^27^. VEMP in this study was evaluated on a qualitative level (presence/absence) with a potential underestimation of clinical conditions characterized by vestibular weakness without failure. However, a motor proficiency delay has been shown in children with extensive vestibular loss or vestibular areflexia^9^. Thus, a VEMP failure could represent a marker of vestibular-related motor delays.

### Study limitations

A major drawback of this study was the high refusal rate (40%), which may affect the inference of the results. Hearing screening can be a distressing moment for the parents, who struggle between hoping for screening dismissal and anxiety anticipating hearing loss confirmation. Such a distressed state, in our opinion, was the most plausible justification for the observed refusal rate. However, the collected sample retains a certain degree of population representativeness, considering the equal proportion of subjects from the referral group and from the at-risk group (48% vs 52%, respectively): in other words, the high refusal rate did not shift the sample distribution towards less severe clinical conditions (refer), returning a weighted spectrum of newborns´ clinical conditions similar to that commonly observed in newborn hearing screening programmes.

## Conclusions

VEMP has shown a high level of feasibility when used alongside the regional newborn hearing screening program. This result supports the use of VEMP as a vestibular screening tool in infants. Further studies are needed to confirm the diagnostic accuracy of VEMP as a form of vestibular screening in children. However, this pilot study has shown that it is possible to adapt a commercially available VEMP device for the large-scale assessment of vestibular function in infants.

## Supplementary information

Supplementary information

## Data Availability

Data treated in this study are available in digital format.
