# Supplementary material for: Methodological aspects of testing vestibular evoked myogenic potentials in infants at universal hearing screening program
Source: Sci Rep. 2019 Nov 21;9:17225. doi: 10.1038/s41598-019-53143-z (PMC6872559; doi:10.1038/s41598-019-53143-z)
Supplement: Supplementary file 1 — Supplementary information [file 41598_2019_53143_MOESM1_ESM.pdf]

Methodological aspects of testing vestibular evoked myogenic potentials in infants at universal hearing screening program.

Luca Verrecchia MD Ph*[1 2], Niki Karpeta MD[1 2], Magnus Westin[1], Ann Johansson[1],Sonny Aldenklint[1], Krister Brantberg MD PhD[1 §], Maoli Duan MD PhD[1 2].

[1] Audiology and Neurotology Unit

Ear Nose and Throat Patient Area

Trauma and Reparative Medicine Theme

Karolinska University Hospital

Stockholm, Sweden

[2]Division of Ear, Nose and Throat Diseases

Dept of Clinical Science, Intervention and Technology

Karolinska Institutet

Stockholm, Sweden

§ currently retired

* corresponding author:

Luca Verrecchia, MD PhD

Division of Ear, Nose and Throat Diseases

Dept of Clinical Science, Intervention and Technology

Karolinska University Hospital, Huddinge, B61

141 86 Stockholm, Sweden

e-mail: luca.verrecchia@ki.se

tel: +46-8-58581565

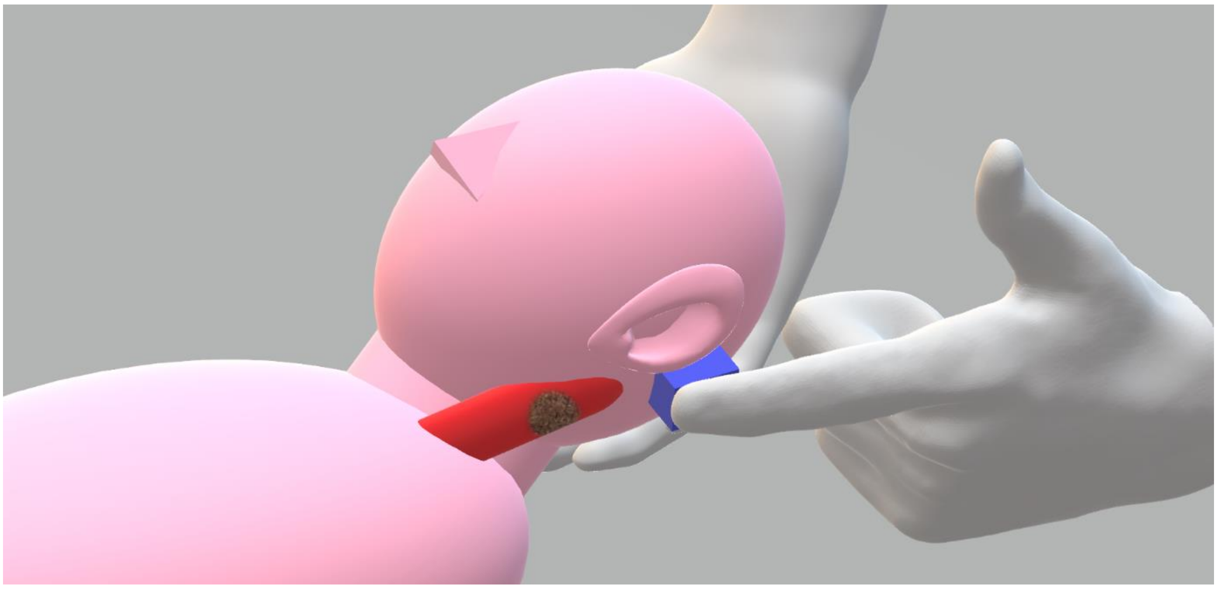

figure 1. A schematic representation of VEMP recording setting in infants. The child is assisted with hand support in the effort to elevate the head in supine position. In this way the contraction of the sternocleidomastoideus muscle(s) (red in picture) is facilitated. The bone transducer (blue in picture) is hold in contact with the mastoid with a latero-medial digital pressure. The muscle activity level (EMG) is detected by the skin electrode (brown in picture) placed upon the sternocleidomastoideus muscle. The EMG level is monitored on screen. The operator can modify the muscle activity level by modulating the head support and following the EMG monitor on screen.

Examples of VEMP recordings. On the Y axis the corrected amplitude and labels indicating the stimulation level (bone conducted tone bursts, 50 dB nHL), the recording side (L: left; R: right) and the trial number (1,2,..). On the X axis the time in ms. P1 and N1 deflections are marked on the recording trials used for calculation. In captures: the description of the cases, the corrected amplitudes (corrAMP) and the prestimulus EMG -mean (SD)- for the marked trial (EMG). Further information in the main text.

CASE 1

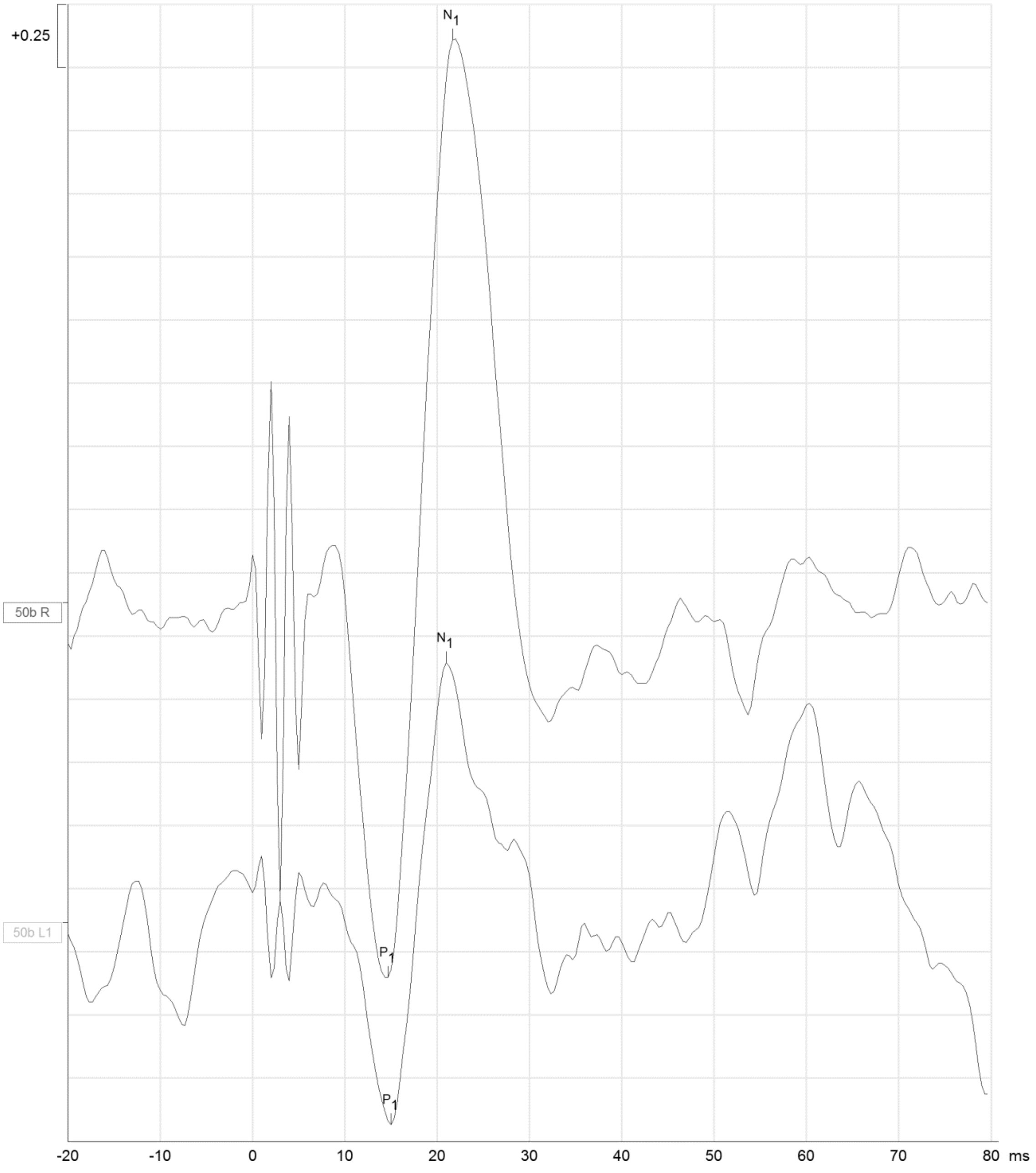

Figure 2. A normal VEMP response of a newborn/infant aged 2 months with normal hearing at screening. Right: corrAMP= 3,71; EMG=50(21) µVolts; Left: corrAMP=1,83 EMG =40(19) µVolts.

CASE 2

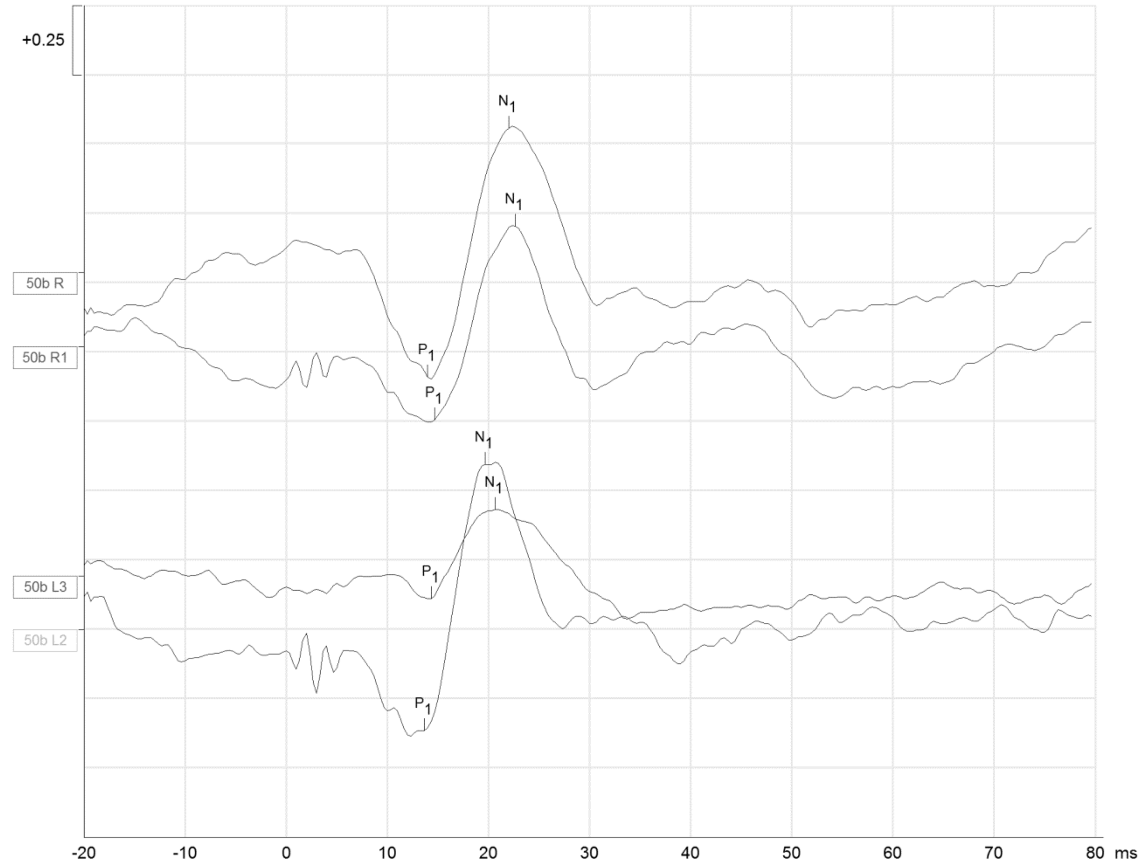

Figure 3. VEMP recording obtained in a female newborn aged one month. VEMP was reproducible bilaterally, but the hearing screening revealed a severe bilateral hearing loss. Further investigations confirmed a profound hearing loss related to pathogenic JBG-2 mutation coding for Connexin 26. Right: corrAMP=0,90; EMG =56(20) µVolts -referred to trial R. Left: corrAMP=0,93; EMG=57(17) µVolts -referred to trial L3.

CASE 3

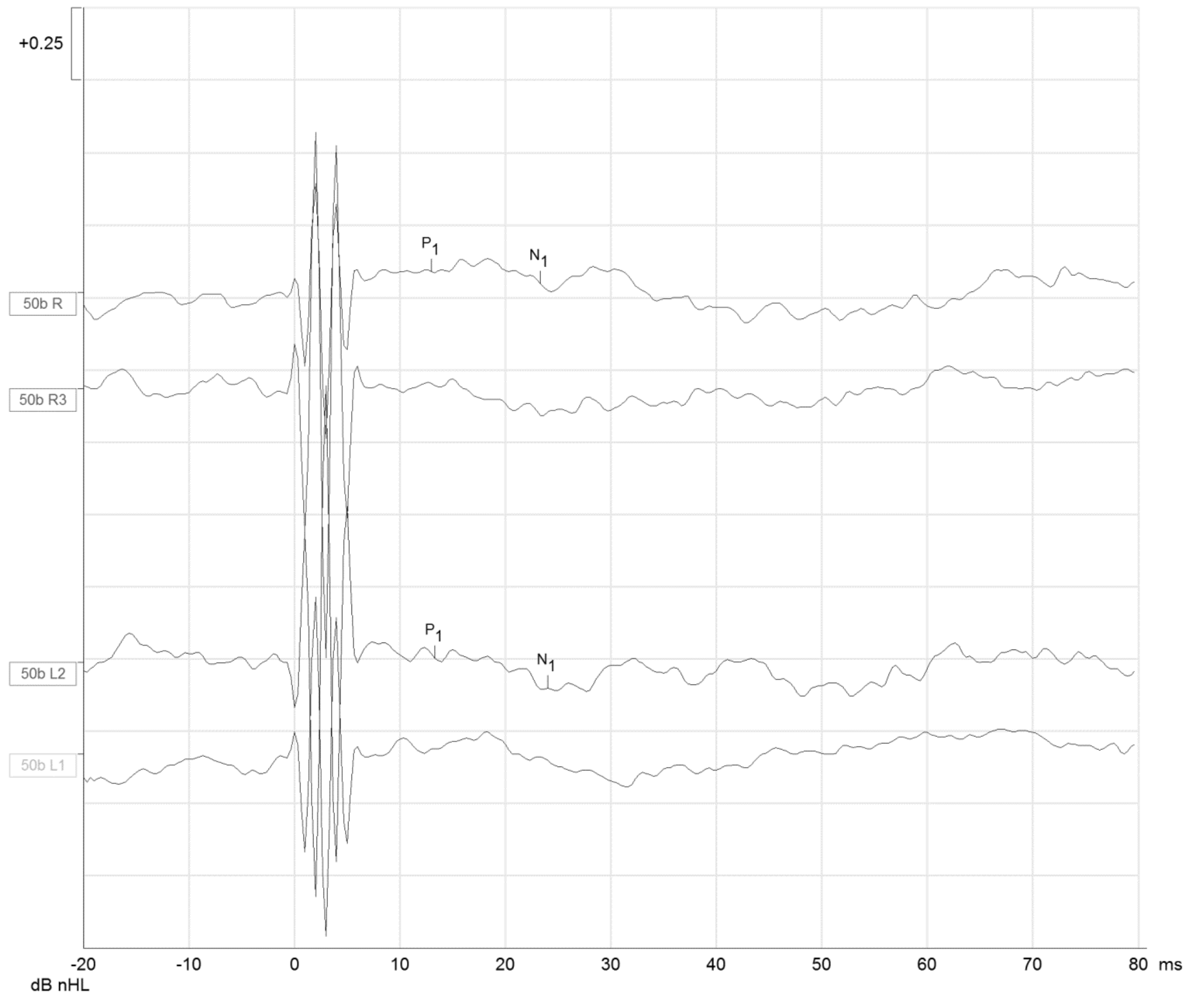

Figure 4. VEMP recording of a female baby aged 6 months with a severe bilateral hearing loss at the third step hearing screening. No VEMP response is appreciable at two test repetitions on the left and the right sides. This child was affected by bilateral agenesis of the audiovestibular nerves as confirmed by a magnetic resonance. Right: corrAMP=0,04; EMG=94(37) µVolts. Left: corrAMP=0,11; EMG=96(45) µVolts.

CASE 4

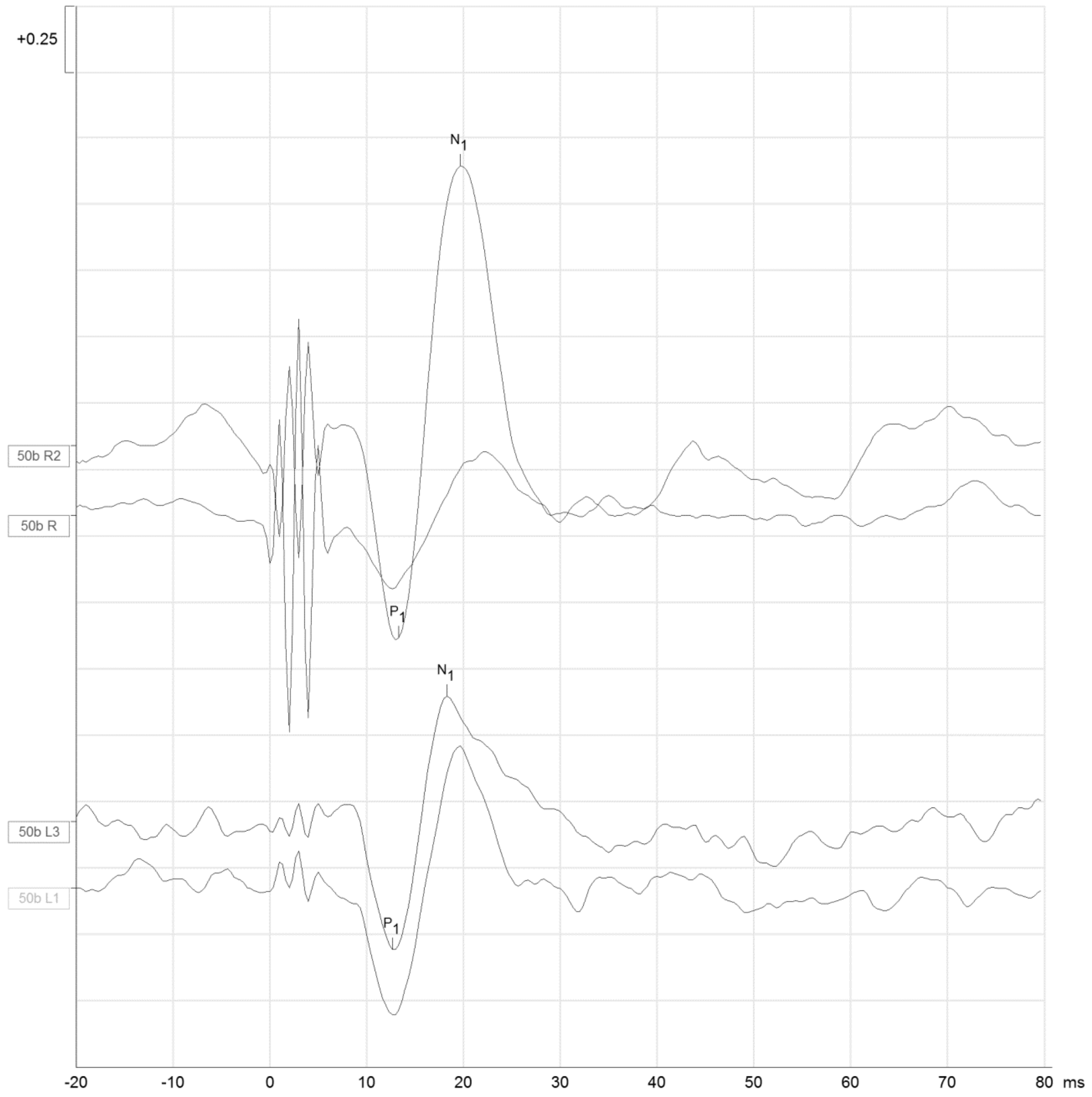

Figure 5. A normal VEMP response at both sides in a female newborn aged 3 months that had suffered severe asphyxia at birth. The hearing result was also normal at the second step of hearing screening program. Right: corrAMP=1,78; EMG=126(75) µVolts. Left: corrAMP=0,96; EMG=137(77) µVolts.

CASE 5

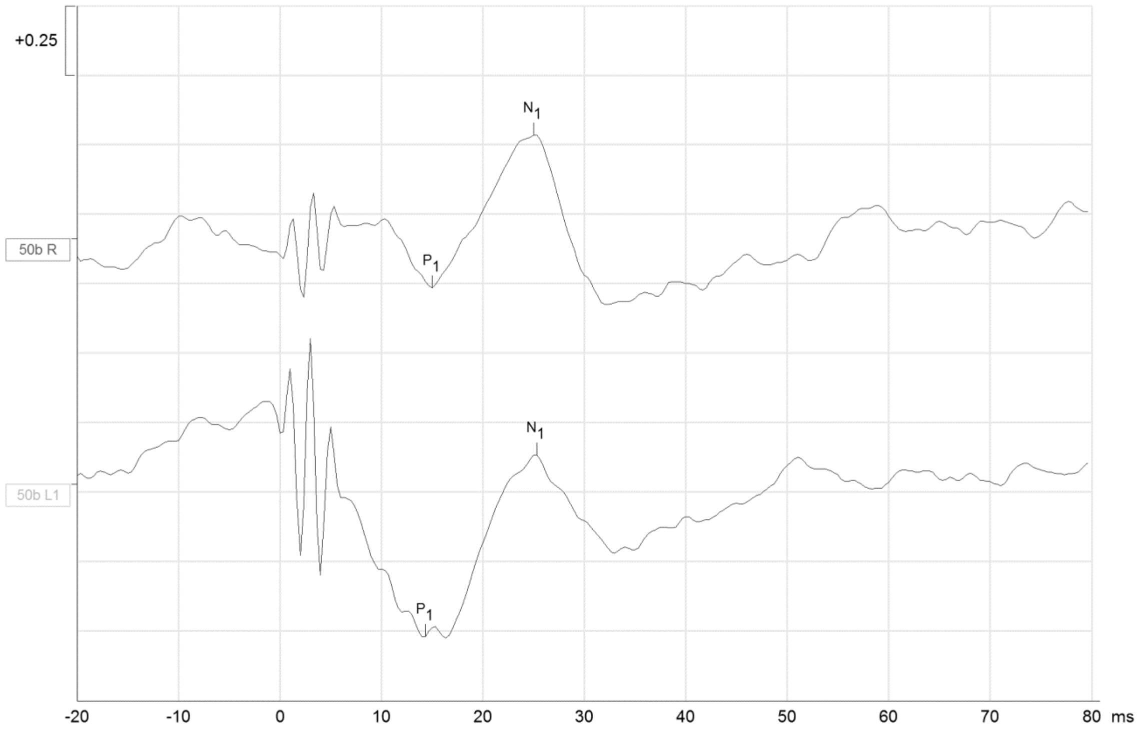

Figure 6. VEMP recording of a newborn with Down syndrome aged one month. HL was recorded on the left side at the second step hearing screening, associated to effusive media otitis. Right: corrAMP=0,55; EMG=113(35) µVolts. Left: corrAMP=0,66; EMG=122(52) µVolts.
